# Autophagy-Lysosomal Pathway as Potential Therapeutic Target in Parkinson’s Disease

**DOI:** 10.3390/cells10123547

**Published:** 2021-12-15

**Authors:** Srinivasa Reddy Bonam, Christine Tranchant, Sylviane Muller

**Affiliations:** 1 Institut National de la Santé et de la Recherche Médicale, Centre de Recherche des Cordeliers, Equipe-Immunopathologie et Immunointervention Thérapeutique, Sorbonne Université, Université de Paris, 75006 Paris, France; 2 Service de Neurologie, Hôpitaux Universitaires de Strasbourg, 67000 Strasbourg, France; Christine.Tranchant@chru-strasbourg.fr; 3 Institut de Génétique et de Biologie Moléculaire et Cellulaire (IGBMC), INSERM-U964/CNRS-UMR7104/Université de Strasbourg, 67400 Illkirch, France; 4 Fédération de Médecine Translationnelle de Strasbourg (FMTS), Université de Strasbourg, 67000 Strasbourg, France; 5 CNRS and Strasbourg University, Unit Biotechnology and Cell Signaling/Strasbourg Drug Discovery and Development Institute (IMS), 67000 Strasbourg, France; 6 University of Strasbourg Institute for Advanced Study (USIAS), 67000 Strasbourg, France

**Keywords:** autophagy, lysosomes, neurodegenerative disease, Parkinson’s disease, autoimmunity

## Abstract

Cellular quality control systems have gained much attention in recent decades. Among these, autophagy is a natural self-preservation mechanism that continuously eliminates toxic cellular components and acts as an anti-ageing process. It is vital for cell survival and to preserve homeostasis. Several cell-type-dependent canonical or non-canonical autophagy pathways have been reported showing varying degrees of selectivity with regard to the substrates targeted. Here, we provide an updated review of the autophagy machinery and discuss the role of various forms of autophagy in neurodegenerative diseases, with a particular focus on Parkinson’s disease. We describe recent findings that have led to the proposal of therapeutic strategies targeting autophagy to alter the course of Parkinson’s disease progression.

## 1. Introduction

**Figure 1:**
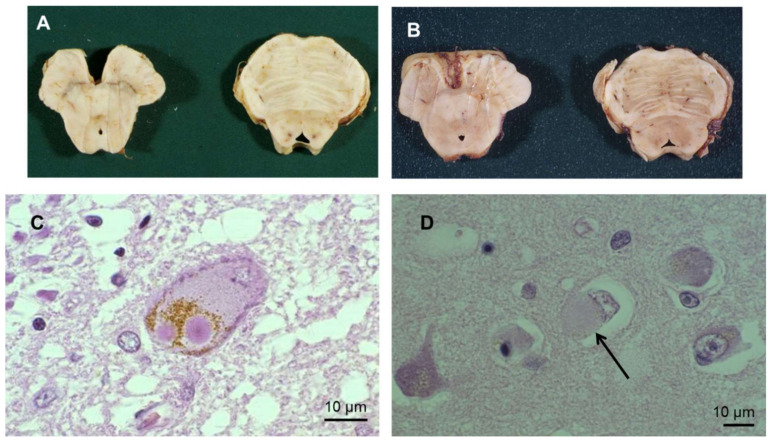
Neuropathological findings in Parkinson’s disease. (**A**,**B**) *Post-mortem* mesencephalon and pons from a control patient (**A**) and from a patient with PD (**B**): SN appeared paler in B due to dopaminergic denervation. (**C**), SN, H&E staining (×250). (**D**): H&E staining (×250) of LB in a cortical neuron. The black arrow shows a LB. Abbreviations not described in the text: H&E, hematoxylin and eosin.

Although some elements of Parkinson’s disease (PD) were described a very long time ago, the first clear medical description of this disease was published in 1817 by James Parkinson [1]. Since then, substantial efforts have been made to understand the underlying pathogenesis and pathological elements of this complex disease, in terms of neuropathological and anatomo-pathological changes [2,3,4,5]. PD is a multifactorial disease with heterogeneous causative factors, including genetic, environmental, molecular and cellular components. PD is characterized by a broad spectrum of motor and non-motor signs and symptoms. They include rest tremor, bradykinesia, postural instability/unsteady gait, rigidity, alongside psychiatric disorders, sleep disorders, dysautonomic disorders, pain, anosmia, and cognitive disorders. Motor signs result primarily from the loss of dopaminergic (DA) neurons in the *substantia nigra pars compacta* (SNpc) and intracellular inclusions of aggregated and misfolded α-synuclein (α-syn), encapsulated or not in Lewy bodies (LB) and Lewy neurites (LN) in the neurons [3,6] (Figure 1; see Appendix A for definition).

The symptoms of PD develop gradually with age. They can start with a slight tremor in one hand and a feeling of stiffness in the body; bradykinesia is frequent. Recent studies confirm that more than 3% of the general population from 65 years of age are affected by PD. In 5%–10% of cases, however, symptoms of PD appear earlier; this is referred to as young-onset PD (YOPD). Men are 50% more likely to develop PD than women, but the risk for women appears to increase with age. 

The root cause of PD remains largely unknown. Some cases of PD have been linked to genetic mutations, but clear hereditary causes of this disease are difficult to establish. Indeed, only 15% of patients with PD have a family history of the disease. Some genes have been associated with distinct, typical or rarer forms of the disease, which include juvenile or adult-onset, early or late, autosomal recessive, dominant or X-linked forms [4,7,8,9]. Causative risk factors associated with particular ethnic groups have also been identified. The genes most frequently linked to PD include *GBA, LRRK2*, *PRKN, SNCA, ATP13A2*, *ATP10B, DJ-1*, *DNAJC6*, *FBXO7*, *HTRA2*, *MAPT*, *PINK1*, *PLA2G6*, *VPS35*, and *VPS13C* [4,7,8,9,10,11,12,13]. The majority of these genes encode proteins that are linked either directly or indirectly to quality control mechanisms that are vital in maintaining cell homeostasis, vesicular transport pathways, autophagy processes, and the endo-lysosomal system. Other genetic alterations have also been associated with PD, including epigenetic changes, such as DNA methylation, chromatin remodeling, histone modifications, microRNAs and long non-coding RNAs [4,14].

## 2. Pathogenesis and Pathology

Clinicopathological studies reveal slow progression of PD from the ventrolateral region of the SNpc, with later spread to other brain regions [15]. Clinical symptoms of PD become detectable when the degeneration of the DA neurons progresses within the SNpc. LBs are observed at sites of neuronal damage (Figure 1). In normal physiology, the α-syn deposited in these structures performs central functions in endocytosis; vesicle trafficking; synthesis, storage and release of dopamine; Ca^2+^ homeostasis; microtubule dynamics; and other processes [16]. Thus, neuronal activity is completely dependent on α-syn, and also on mitochondrial homeostasis. Although α-syn is predominantly present in cytosolic eosinophilic LBs, it has also been detected in mitochondria, lysosomes, and other organelles in *post-mortem* PD brains. The presence of LBs in the peripheral, enteric, and central nervous systems (CNS) has been implicated in both motor and non-motor symptoms of PD [17,18]. Point mutations in the α-syn sequence or other pathological insults leads to the formation of oligomers, which may then group together as larger aggregates. These aggregates can alter numerous cellular and molecular pathways in neurons—in particular involving autophagy and proteasomal processes, such as mitochondrial function, vesicle trafficking, organelle, and protein degradation—all of which lead to neurodegeneration. Subsequently, as a result of the neurodegeneration, α-syn aggregates are deposited in the SN, where they activate microglia [19]. This uncontrollable activation can generate pro-inflammatory signals [20], which may lead to further neurodegeneration when a critical threshold is reached.

### 2.1. Neuropsychiatric Manifestations of PD

No specific test exists to diagnose PD. Consequently, diagnosis is based on medical history, a review of signs and symptoms, and a neurological and physical examination (Box 1). Motor signs of PD usually begin around 60 years of age [21], but YOPD is not rare, particularly in some hereditary forms [22]. Unilateral or asymmetric bradykinesia and/or rest tremor are the first symptoms of the disease [23]. Rest tremor is present in relaxed muscles and disappears during action and sleep. It may be increased by mental calculation. Bradykinesia, defined by slowness of movement and decreased amplitude or speed, leads to difficulties with repetitive movements, micrography, small-step gait, speech difficulties (hypophonia and dysarthria), which will emerge as the disease evolves. Rigidity may cause pain and contribute to postural deformity (thoracolumbar spinal flexion). Progression is slow with bilateral extension of akinesia, tremor, and hypertonia, followed by postural instability, freezing of gait, falls, and in some patients, camptocormia. Some non-motor signs (premotor) may occur several years before the first motor symptoms; these include depression, hyposmia, constipation, or rapid-eye-movement sleep disorders [24]. Anxiety and apathy may be present from the onset of PD, whereas severe dysautonomia (orthostatic hypotension, urinary dysfunction due to detrusor hyperactivity), sleep fragmentation, cognitive disorders (dysexecutive disorders), and hallucinations emerge later, and will contribute to loss of autonomy [24,25].

Box 1 **Diagnosis of PD.**

Parkinsonian syndrome, which may have numerous causes, is defined by the presence of bradykinesia and at least two features among rest tremor, plastic hypertonia, or both. Among parkinsonian syndromes, diagnosis of PD, based on the Movement Disorder Society international criteria [23], rests on

- at least two supportive criteria (e.g., clear positive response to L-dopa, induced dyskinesia, rest tremor),
- absence of absolute exclusion criteria (e.g., associated cerebellar signs, vertical ocular palsy, frontotemporal dementia or sensitive signs, no L-dopa response, isolated lower limb parkinsonism, treatment with dopamine receptor blockers), and
- absence of red flags (e.g., rapid progression with, over the 5 first years, wheelchair use, bulbar dysfunction, severe dysautonomic dysfunction, recurrent falls during the first 3 years, inspiratory respiratory dysfunction, associated pyramidal signs or muscle contractures (neck, hands; feet), bilateral and symmetric evolution, absence of dopamine sensitivity and /or evolution, absence of non-motor signs).

### 2.2. Current Treatments for PD and Clinical Management

Symptomatic treatment is the only clinical option currently available [26], with therapies aiming to compensate for the dopaminergic deficit. Dopaminergic drugs (levodopa associated with dopa-decarboxylase inhibitor, dopaminergic agonists, or monoamine oxidase-type B inhibitors), used individually or in poly-therapy regimens, are very efficient during the early stages of the disease. However, management becomes more difficult as the years progress. Indeed, dopaminergic treatments, which improve motor signs, can have very disabling complications. A wearing-off phenomenon (end of dose failure) and dyskinesia occur after several years of levodopa treatment [27]. Impulse control disorders (pathological gambling or shopping, hyper sexuality; [28], hallucinations, or psychosis may also complicate dopaminergic treatments, and are more frequently encountered with dopamine agonists [29]. Other treatments, including catechol-O-methyltransferase inhibitors to treat motor fluctuations, or amantadine for dyskinesia [26] can be used later as the disease progresses. Second-line treatment (continuous subcutaneous infusion of apomorphine, continuous jejunal administration of duodopa gel, bilateral subthalamic nuclear stimulation) are proposed when motor fluctuations and dyskinesia become significant [30]. These treatments aim to achieve stable striatal dopaminergic stimulation but have no impact on disease progression. Furthermore, some axial symptoms (dysarthria, postural instability) are not dopa-sensitive, and clinical management of non-motor symptoms remains difficult [31,32].

Decades of investigations have led to the development of therapeutic strategies, which have undoubtedly improved quality of life for patients. However, slowing the disease’s progression still remains a challenge, and a persistent priority [33], and novel disease-modifying approaches are eagerly awaited [3,34]. Although the functions of the proteasome and of autophagy, including macroautophagy and chaperone-mediated autophagy (CMA), have long been known to contribute to α-syn clearance [35,36], dysregulation of these processes remains poorly understood in PD. Several gene mutations and alterations to proteins involved in PD are closely linked to autophagy, particularly mitophagy and autophagy-lysosomal pathways. In this review, we focus on the involvement of autophagy in PD, we comment on the major unanswered questions in the field and propose new directions for possible therapeutic interventions targeting autophagy pathways.

## 3. Autophagy

**Figure 2:**
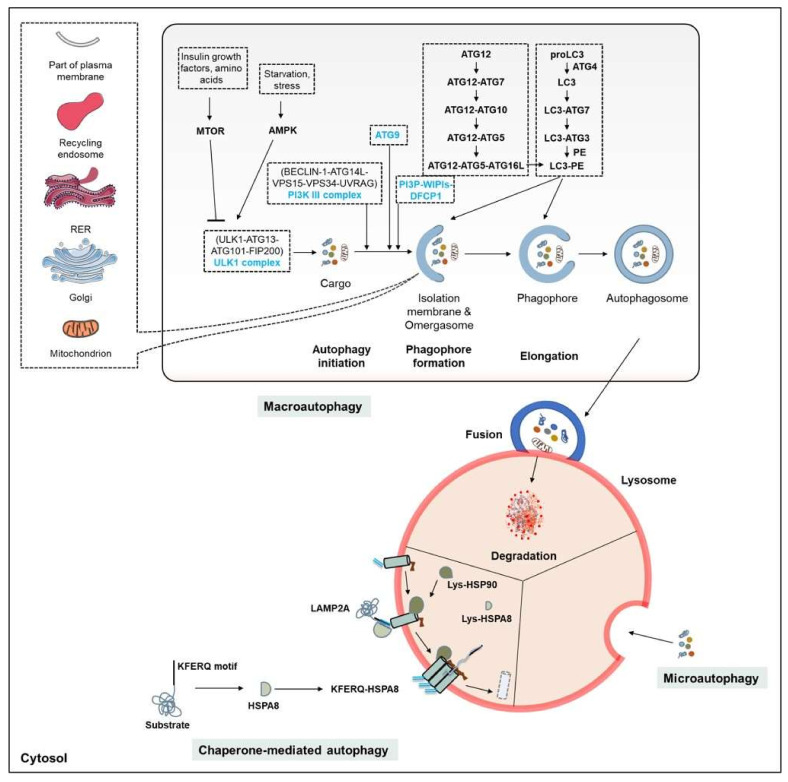
The three main types of autophagy. *Macroautophagy:* schematic representation of the sequential steps: initiation/nucleation, phagophore formation, phagophore elongation, closure (autophagosome), fusion, and degradation. Under nutrient/energy-rich conditions (insulin growth factors, amino acids) MTOR inhibits autophagy initiation by regulating the ULK1 complex. In contrast, AMPK activates autophagy in response to stress or starvation. *CMA:* process that degrades substrates containing a KFERQ-motif (or CMA-targeting recognition motif). The chaperone HSPA8 forms a complex with other co-chaperones (HSP90, HSP40, HSP70-interacting protein/HIP and HSP70-HSP90 organizing protein/HOP) that not only acts as a catalyst allowing unfolded proteins to refold, but also mediates delivery of proteins to the lysosome via the LAMP2A receptor at the lysosomal membrane. *Microautophagy:* in this process, the cytoplasmic content destined for autophagy is directly engulfed into the lysosome following formation of an invagination. Abbreviations not described in the text: DFCP1, double FYVE domain-containing protein 1; RB1CC1/FIP200, FAK-family interacting protein of 200 kDa; RER, rough ER; UVRAG, UV resistance-associated gene; WIPI, WD repeat domain phosphoinositide-interacting.

Autophagy is a major intracellular degradation system by which cytoplasmic materials are delivered to the lysosome for degradation. Based on the route by which content is delivered to lysosomes, several forms of autophagy have been defined. These different forms also have varying degrees of selectivity for targeted cargos (Table 1; Figure 2). The three main types of autophagy process are macroautophagy, CMA, and microautophagy/eMi. Whatever the delivery route, the main role of these processes is to degrade unwanted material that is defective, may be toxic, or has been produced in excess, and thus to maintain cell homeostasis.

### 3.1. The Autophagy Machinery

The mechanisms of autophagy have been thoroughly investigated and were reviewed in detail by numerous authors [61,62,63]. The general features of the three pathways are presented in Figure 2. Recent advances related to canonical and non-canonical autophagic processes—especially in mammalian systems—have added to our understanding of the mechanisms that may play important roles in neurodegenerative diseases such as PD. Nevertheless, many of the molecular discoveries upon which our current understanding of the regulation of autophagy is based emerged from analyses involving yeast. In cells, the three forms of autophagy coexist, and play vital roles in maintaining cellular homeostasis. However, the large majority of results available in this field relate to macroautophagy. This process has been divided into the following steps: nucleation, elongation, autophagosome formation, autophagosome-lysosome fusion, and degradation (Figure 2). Each step is finely genetically-regulated and plays its own specific role in maintaining the dynamic nature of the process. For example, a number of conserved autophagy-related proteins act in a hierarchical manner to mediate autophagosome formation. Upon upstream induction, the autophagy machinery comes into contact with the isolation membrane/phagophore. The early origins and definitive complex source [endoplasmic reticulum (ER), Golgi complex, endosomes, and mitochondria] of the nascent isolation membrane remains a matter of debate [64]. An ultrastructural study involving electron microscopy experiments confirmed that a specialized subdomain of the ER contributes to phagophore generation [65]. About 40 autophagy-related (ATG) proteins have been identified as involved in this dynamic process, they are hierarchically organized, starting from the initiation of the process and progressing through to the maturation of autophagosomes. These proteins work together in several functional complexes, notably (i) the Unc-51-like kinase 1 (ULK1)/ATG1 kinase complex; (ii) the class III phosphatidylinositol (PI) 3-kinase complex; (iii) the PI(3)P-binding ATG2-ATG18 complex; (iv) the two conjugation systems (ATG12 conjugation system and microtubule-associated protein 1A/1B-light chain 3 (MAP1LC3)/ATG8 conjugation system); and (v) the fusion machinery (Figure 2). 

In fact, several so-called ATG proteins have alternative functions beyond autophagy [66]. Thus, for example, MAP1LC3 lipidation (a mechanism that has long been used to assess autophagic activity [67,68]) is also involved in non-autophagic cellular mechanisms such as phagocytosis, LAP, micropinocytosis, or viral infection. These processes are known as non-canonical autophagic processes [69]. In these non-canonical processes, the functions of which are still incompletely characterized [70,71] MAP1LC3 conjugates to single membranes (single membrane ATG8 conjugation, SMAC), and cytosolic constituents are not delivered to the lysosome [72].

### 3.2. Neuronal Autophagy Contributes to Neuronal Physiology

There is compelling evidence to support the idea that neuronal autophagy plays a decisive role in several aspects of neuron development and in preserving neuronal activity [73,74,75]. In post-mitotic cells like neurons, autophagy is especially important for survival and homeostasis, because these cells cannot eliminate accumulated toxic substances and damaged organelles during cell division. Autophagy, as well as the proteasomal system [76], is therefore one of the vital quality control mechanisms that ensure the longevity of neuronal cells. Presynaptic autophagy in the axon terminal is also essential for synaptic maintenance and plasticity [77].

Among nerve cells, only cortical neurons, Purkinje cells, and hypothalamic neurons can increase their autophagosome content upon a stimulus. The precise reasons for this niche mechanism are currently unknown [62,78]. One possible explanation is trivial and related to the fact that, like for some other cell types, measuring autophagy in neurons, especially in the brain, remains challenging [79,80]. Alternatively, because nerve cells are terminally differentiated—with a lower regenerative capacity than other cells—they are less autophagic. However, studies on brains from autophagy-deficient mice provided evidence that sequestosome-1 (SQSTM1)/p62 protein and polyubiquitinated proteins accumulate in most neuronal cells [81]. In contrast, SQSTM1 deficiency does not result in a complete lack of autophagy. It appears therefore that the autophagosome content depends on the type of cells and the type of stressor.

### 3.3. Autophagy and Neurodegenerative Diseases

As introduced above, to prevent neuronal and synaptic dysfunction, neurons have evolved mechanisms to remove toxic and defective components and organelles. These mechanisms are essential to maintain a high degree of neurotransmission and the integrity of the functional proteome in neurons. Autophagy is central to this protective system. Age-related functional loss of autophagy makes the neurons more vulnerable to stress and can lead to cell death [82]. Pathological disruption of autophagy pathways can also result in neurodegenerative disorders that may or may not be linked to ageing.

Compromised autophagy has been documented in many neurodegenerative diseases, including PD, Alzheimer’s disease (AD), Huntington’s disease (HD), and amyotrophic lateral sclerosis (ALS) (for comprehensive reviews, see [63,83,84]). In investigating the mechanisms linking autophagy to these diseases, it has been observed, for example, that mice specifically deficient for *Atg5* in neural cells develop progressive deficits in motor function while also accumulating cytoplasmic inclusion bodies in neurons [85]. Similarly, in mice deficient for *Atg7*, *Atg5*, or *Ambra1*, ubiquitin was found to accumulate in the CNS, and cytoplasmic inclusions were associated with motor dysfunctions, and neuronal tube defects in mouse embryos [86]. Mutation of genes linked to autophagic processes—for example, SQSTM1, optineurin/OPTN, E3 ubiquitin ligase PARKIN/PRKN, PINK1, TBK1—have also been implicated in many neurodegenerative diseases. In particular, defects in mitophagy that are also seen in organ-specific and systemic inflammatory diseases, have been documented in neurodegenerative diseases [87]. In addition to these genetic mutations, significant alterations to protein expression have been linked to neurodegenerative diseases. For example, abnormal expression of the protein glandular epithelial cell 1 (GABARAPL1/GEC1) has been associated with neurodegenerative diseases [88].

### 3.4. Autophagy and Parkinson’s Disease

**Figure 3:**
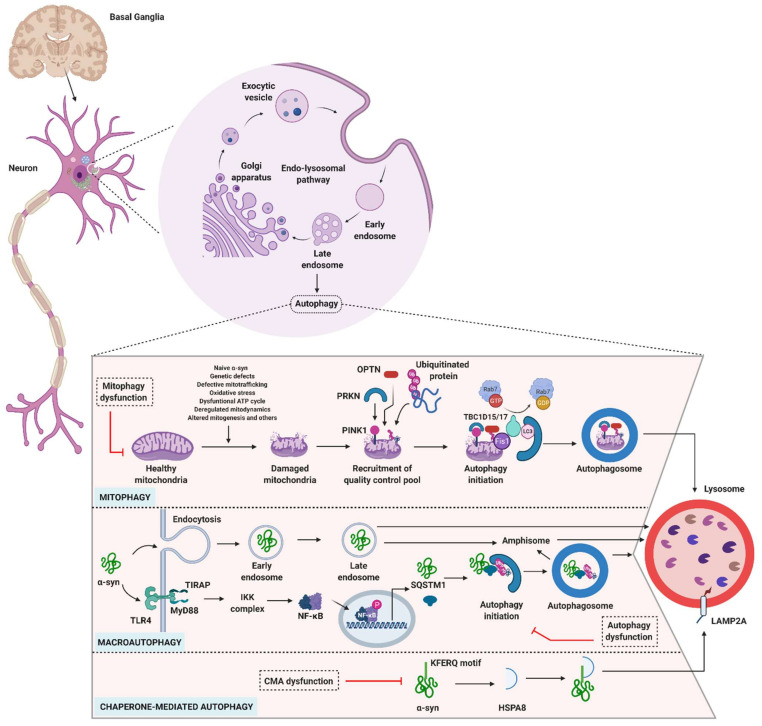
Autophagy impairment in PD. Impaired forms of autophagy have been observed in PD. Genetic mutations of α-syn are linked to impairment of the autophagy process. Many factors, such as genetic factors, defective mitochondrial trafficking, oxidative stress, dysfunctional ATP cycle, deregulated mitochondrial dynamics, and altered mitogenesis perturb healthy mitochondria. Damaged/dysfunctional mitochondria allow PINK1 to recruit PRKN, which in turn activates other essential proteins, such as OPTN and ubiquitin, Rab7 and others thus to initiate a quality control process, i.e., mitophagy. The function of the Rab7 is regulated by the TBC1D15/17 (belong to the TBC family with Rab-GAP functions), which is also regulate the shaping and target functions of isolation membrane by cross-linking with Fis1 and MAP1LC3B. The sequential steps of mitophagy are: formation of the phagophore, maturation into the mitoautophagosome, and fusion of the mitoautophagosome with the lysosome. Conventional autophagy also plays an essential role in (both naïve and aggregated) α-syn degradation. α-syn selectively binds to the pathogen-recognition receptor, TLR-4, which activates the downstream signaling pathway following NF-κB activation, to stimulate SQSTM1/p62 production. The SQSTM1 produced binds to the internalized α-syn and initiates the autophagy process. Dysregulation of the autophagy process leads to the accumulation of α-syn alongside SQSTM1. Apart from mitophagy and macroautophagy, CMA also selectively degrades α-syn, which contains a KFERQ-like motif. Selective CMA inhibition or altered CMA functioning affect α-syn degradation. Abbreviations not described in the text: Fis1, Mitochondrial fission 1 protein; GAP, GTPase-activating proteins; IKK, IκB kinase; MyD88, myeloid differentiation protein 88; Rab, Ras superfamily of small G proteins; TBC, Tre-2/Bub2/Cdc16; TIRAP, Toll-interleukin 1 receptor adaptor protein.

Among the pathological hallmarks of PD are LBs that contain abnormally aggregated α-syn protein. Mutations or triplication of the gene encoding α-syn (*SNCA*) are rare, but are clearly involved in the initiation and progression of PD. Interestingly, any failure affecting one of the components of the degradative process, either directly or indirectly, impairs the other autophagy processes. The ubiquitin-proteasome system (UPS) is known to be the primary degradative pathway for monoubiquitinated α-syn, whereas the macroautophagy pathway degrades deubiquitinated α-syn [89,90]. In PD, therefore, both mitochondria and lysosomes play crucial roles (Figure 3).

#### 3.4.1. Role of Mitophagy in PD

As an energy producing organelle, the mitochondrion is central to several neurodegenerative diseases, including PD [91,92,93,94,95]. Multiple investigations revealed that genetic mutations associated with PD (e.g., *PRKN*, *PINK1*, and others) are also closely linked to mitochondrial defects, including defects in mitophagy (Table 2) [96]. The type of damage caused to the mitochondria naturally depends on the type of α-syn (forming aggregates or not, produced from mutated or native forms of *SNCA*). Further studies confirmed that α-syn affects the interaction of the mitochondria-associated membrane with the ER. This interaction plays a pivotal role in regulating Ca^2+^ signaling and apoptosis. In addition, abnormal α-syn interferes with the peroxisome proliferator-activated receptor gamma coactivator 1-alpha, which plays a crucial role in mitochondrial biogenesis and in apoptosis. Mitochondrial dysfunction with the involvement of factors related to α-syn has been comprehensively discussed elsewhere [9,97,98].

Defects in the mitophagy pathway, especially PARK2 (*PRKN* mutations) and PARK6 (*PINK1* mutations), have been proposed as a major cause of familial PD. In healthy conditions, PINK1, which localizes to the mitochondrion, is translocated into the mitochondrial inner membrane where it is degraded. Under certain unknown conditions, mitochondria become damaged and lose membrane potential (Figure 3). This leads to PINK1 activation and recruitment of PRKN, which helps to induce mitophagy while acting on other mitochondrial membrane proteins [OPTN and nuclear dot protein 52-kDa (NDP52)] [62,119,120,121,122,123,124]. *PRKN* mutations are the most frequent cause of autosomal recessive YOPD, followed by mutations in PINK1. Alongside its role in mitophagy, PRKN plays an essential role in lipid processing and the ubiquitination of the GTPase Rab7, which regulate lysosomal dynamics [125,126,127,128]. PRKN deficiency results in DA neuronal degeneration in mice, and embryonic fibroblasts derived from PINK1-deficient mice show lysosomal dysfunction [129]. In addition, mutations in *PINK1* and *PRKN* lead to defects in the mitophagy process [62]. However, studies have yet to explain why PRKN is not recruited to mitochondria in DA neurons under depolarized conditions [130]. A consequence of mitophagy dysfunctions in neurons is uncontrolled stress (i.e., generation of reactive oxygen species), which causes neuronal cell death. In line with this effect, targeting mitophagy defects may be beneficial in PD. It has been shown, for example, that an inhibitor of the mitochondrial deubiquitinase USP30, which negatively regulates PRKN-mediated mitophagy, selectively increases mitophagic flux, thus it could be of interest for the development of novel therapeutic approaches [131,132]. 

In addition to the major effect of *PINK1* and *PRKN* mutations, *SNCA* mutations have been studied in the context of mitophagy. α-syn interacts with the Miro proteins (outer mitochondrial membrane adapter proteins, useful in mitochondrial motility) and interferes with the Miro degradation process, which is an essential step in the mitophagy process [133]. Studies in mice and yeast harboring mutations in *SCNA* confirmed the role of α-syn in neuronal death, via mitochondrial dysfunction [134,135].

The transcription factor myocyte enhancer factor 2D (MEF2D) is another essential mitochondrial regulator (Table 2). It is a central factor in the transmission of extracellular signals and activation of genetic programs in response to a wide range of stimuli in several cell types, including neurons. MEF2D is a critical regulator of IL-10 gene expression, involved in negative control of the microglial inflammatory response, and preventing inflammation-mediated cytotoxicity [136]. Reduced MEF2D expression has been directly linked to reduced levels of nicotinamide adenine dinucleotide dehydrogenase 6 (NADH), a component of mitochondrial complex I. *Post-mortem* analysis of brain samples from PD patients revealed reduced levels of both MEF2D and NADH [137].

A number of other genetic mutations, including deficiencies in mitochondrial apoptosis-inducing factor (AIF) and mitochondrial transcription factor A (TFAM; [138] that perturb endo-lysosomal pathways, also affect mitochondrial physiology and function, leading for example to impaired mitophagy, dysfunctional oxidative phosphorylation, deregulated mitochondrial dynamics, altered mitogenesis, calcium imbalance, altered mitochondrial trafficking, and induction of oxidative stress (Table 2). PRKN-independent autophagy pathways are involved in the selective mitophagy process via receptor-mediated, lipid-mediated, and ubiquitin ligase-mediated pathways [97,139,140]. It is not currently known to what extent these pathways are linked to PD.

#### 3.4.2. Role of Macroautophagy in PD

The involvement of macroautophagy and its defects have been extensively investigated in neurodegenerative diseases and synucleinopathy, with some studies also referring to PD [141,142,143,144]. Accumulating evidence indicates that several components of the macroautophagy pathway are involved in PD (Figure 3). Genetic analyses of PD patients have revealed abnormal expression levels for the genes encoding ATG5, ATG7, ATG12, and MAP1LC3B (Table 2). Two independent exome sequencing studies [145,146] also identified point mutations in the vacuolar protein sorting ortholog 35 (VPS35) gene, causing an autosomal dominant form of PD (PARK17) (Table 2). This monogenic subtype of PD is rare, and the precise role of the *VPS35* mutations (especially VPS35 D620N) is not yet fully understood (discussed in [147]. Nevertheless, this pathogenic effect highlights the role of the retromer, a highly conserved membrane-associated protein complex in which VPS35 is an intrinsic component, in the α-syn degradation pathway [148,149]. Studies on DA neurons lacking the *VPS35* gene show α-syn accumulation and toxicity, in particular loss of mitochondrial fusion and functions [150]. ATG9 trafficking defects (the ATG9 system is required for phagophore expansion) were also associated with mutant VPS35, leading to α-syn accumulation. Increasing VPS35 levels in PD mice rescued α-syn accumulation and induced neuroprotection, demonstrating that regulating VSP35 may be of interest to treat PD [147,151].

Although still a matter of debate, recent data suggest the involvement of more selective autophagy pathways particularly targeting α-syn, that is, synucleinphagy (Table 1) [58]. In Vitro and in vivo studies conducted on microglial cells clearly demonstrated that α-syn was degraded via macroautophagy. Toll-like receptor (TLR)-4 on the microglia recognizes α-syn and activates NF-κB signaling, which in turn alters SQSTM1 transcription. The protein produced, which is present at raised levels, selectively binds to the internalized α-syn leading to its colocalization with autophagosomes. Lack of TLR-4 and SQSTM1 alters autophagy-mediated α-syn degradation [58]. Thus, TLR-4 or SQSTM1 may serve as relevant markers in neurons where α-syn has accumulated.

#### 3.4.3. Role of CMA in PD

In addition to mitophagy and lysosomal macroautophagy, which have relatively well-established roles in α-syn degradation, CMA also appears to be involved in this vital process. CMA is a central process that degrades proteins harboring a specific combination of five amino acids—the “KFERQ-motif” (Figure 2 and Figure 3). This motif allows the chaperone heat shock protein A8 (HSPA8)/cognate 70 kDa protein (HSC70) to bind the substrate protein and direct it to the lysosomal membrane, where it encounters lysosome-associated membrane protein type 2A (LAMP2A), which plays a decisive role. Binding of the complex promotes LAMP2A multimerization to form a membrane-bound higher-molecular–order complex. The substrate protein is then unfolded within this complex and translocated into the lysosome. A lysosome-resident HSPA8 isoform (Lys-HSPA8) contributes to the translocation of the substrate protein across the membrane towards the lysosomal lumen, where it is degraded by acidic hydrolases [36,152,153,154,155]. α-syn contains a KFERQ-like motif and its CMA-mediated degradation is significantly reduced for α-syn constructs lacking the KFERQ-like motif, and by LAMP2A knockdown [152,156].

Interestingly, another protein, leucine-rich repeat kinase 2 (LRRK2)—which is also linked to familial forms of PD (Table 2)—is degraded in lysosomes as part of CMA. In contrast, the most common pathogenic mutant form of LRRK2, G2019S, is poorly degraded by this pathway [154,157]. CMA activity can be modulated by the rate of assembly/disassembly of the translocation complex [40]. In this context, a key finding for PD was the discovery that lysosomal binding of both wild-type and several pathogenic mutant LRRK2 forms was increased in the presence of other CMA substrates. These substrates interfere with the organization of the CMA translocation complex as the enhanced binding inhibits assembly of the CMA translocation complex at the lysosomal membrane. In response to this inhibition, affected cells produced more LAMP2A. A similar feature has been observed in brains from PD patients with *LRRK2* mutations. This mechanism leads to the accumulation of other CMA substrates, including α-syn, that remain bound longer than normal at the surface of the lysosomal membrane awaiting translocation [157]. Thus, in PD, the *SCNA* gene is not alone in contributing to the pathology, and genes involved in the clearance of aggregated α-syn may also be involved [154]. This finding is significant since both mutant α-syn and aggregated α-syn that have escaped autophagic degradation can hamper the CMA process in neurons, leading to neuronal cell death [158].

Several *post-mortem* studies have revealed that the levels of rate-limiting CMA components LAMP2A and HSPA8 are reduced in PD patients, especially in the SNpc [156,159,160]. Interestingly, the oxidized mitochondrial regulator MEF2D described above also binds to HSPA8 and is indirectly involved in CMA [161]. Studies on peripheral leukocytes from patients with sporadic PD revealed that *LAMP2* transcript and protein levels are significantly reduced compared to the levels measured in healthy individuals [162]. Although in the same study, macroautophagy (measured by MAP1LC3II analysis) appeared to be induced, this conclusion would merit more complete independent confirmation, with the addition of measurement of autophagic flux (not performed in the initial study). 

A few other autophagy-related genes and proteins involved in CMA have been associated with PD. For example, the peroxiredoxin-like redox sensor DJ-1 modulates the activity of SQSTM1 and the targeting of ubiquitin-conjugated proteins to macroautophagy under oxidative stress caused by tumor necrosis factor ligand superfamily, member 10 (TNFSF10/TRAIL) [163]. The implication of mutated *DJ-1* (PARK7) in early-onset familial PD is well known (Table 2). DJ-1 is involved in a wide variety of cellular functions, including a role as an antioxidant, chaperone functions, transcriptional regulation, control of mitochondrial Ca^2+^ transients, among others. This protein regulates p53-induced mitochondrial dysfunction, stabilizes mitochondria-associated ER membranes, and interacts with anti-apoptotic proteins [164]. In addition to its effects on mitophagy and lysosomal macroautophagy, DJ-1 also modulates CMA through its interactions with LAMP2A and lysosomal HSPA8 [165]. In particular, it escorts wild-type α-syn for degradation by CMA. Lack of the *DJ-1* gene inhibits CMA activity and α-syn degradation both in vitro and in vivo [165]. In contrast, DJ-1 is stabilized in cortical neurons by a CMA-mediated process that involves LAMP2A [166]. Interestingly, mutated α-syn proteins can escape from CMA-mediated degradation, which once again supports the CMA selective autophagy process.

In addition to impaired autophagy due to CMA-related genes in familial PD, alterations to CMA have also been implicated in sporadic PD, which accounts for the majority of PD cases. The etiology is more complicated to define in this setting and may be related to various factors—including environmental (e.g., pesticides) and cellular (e.g., oxidative stress; see above) stressors. The proposed involvement of CMA malfunction in PD pathogenesis is further supported by age-related changes to the LAMP2A protein itself, which lead to a gradual reduction in CMA and subsequent acceleration of the disease in older patients [154,156,157,167,168].

Recently, several notable data have highlighted various immune alterations underlying that PD is associated to autoimmune features and could be considered as an autoimmune disease [87]. The presence of serum autoantibodies reacting with LAMP2A has not yet been investigated in PD. These autoreactive antibodies have been described recently in the serum of autoimmune patients with lupus and closely related systemic autoimmune diseases [169]. 

#### 3.4.4. Role of Lysosomes in PD

Regardless of the roles played by the different types of autophagy in PD (macroautophagy, CMA and even some forms of secretory autophagy [170]), lysosomes play a central role in α-syn degradation. Alterations to the lysosomal enzyme content (particularly hydrolases) influence the degradation process, leading to the accumulation of protein aggregates which cause neuronal damage [48]. Studies have reported decreased levels of lysosomal hydrolases such as α-mannosidase, β-mannosidase, and β-glucocerebrosidase (GBA) in the cerebrospinal fluid (CSF) from patients with PD. In contrast, in the same patients’ serum the activity of these hydrolases was not significantly altered [171]. The difference in levels of hydrolases in the CSF is now being used for diagnostic purposes [171]. In contrast, the level of α-syn in CSF from PD patients was found to be greatly diminished [172]. These reduced levels are probably due to α-syn accumulation in LBs [172]. Several genes associated with PD are also directly linked to lysosomal functions. As mentioned above, all the autophagy pathways at some point involve lysosomes, as the point to which the material is delivered for processing by various enzymes [48]. Among the enzymes implicated in these processes, cathepsin D is associated with α-syn degradation. Studies on Drosophila have shown that lack of cathepsin D leads to the accumulation of unprocessed substrates in lysosomes and late endosomes [10,148].

GBA, encoded by *GBA1*, is another lysosomal enzyme involved in α-syn degradation. Lack of GBA function or low levels of GBA in the human brain induce the accumulation of an oligomeric form of α-syn [173,174,175], which in turn interferes with the maturation of GBA [173], leading to a vicious cycle. Heterozygous mutations in *ATP10B*, which encodes ATP10B (Table 2), a late endo-lysosomal lipid flippase that translocates the lipids glucosylceramide and phosphatidylcholine towards the cytosolic membrane leaflet, have been implicated in PD [13]. These mutations alter the translocation functions of ATP10B, leading to an accumulation of glucosylceramide which can drive lysosomal dysfunction [13].

ATP13A2 (PARK9) (Table 2) is another lysosomal protein that is important in PD. It is responsible for cation transport in lysosome-like vesicles. Mutations in *ATP13A2* have been observed in early-onset PD [12]. In Vitro studies confirmed that increased ATP13A2 protein levels reduce α-syn-induced toxicity [176,177]. Similarly, reduced levels of α-galactosidase A transcript and protein were observed in peripheral blood mononuclear cells from PD patients [178]. In addition, studies on the brain of PD patients revealed that DA neurons contained numerous lysosome-like vesicles and granules, suggesting that even in the final stages of the disease, these neurons undergo active apoptosis and engage in autophagy processes [179].

Human transmembrane protein 175 (TMEM175), one of the highly expressed genes that encode lysosome-bound K^+^ channels has also been linked to PD pathogenesis. Both in vitro and in vivo studies on neurons confirmed that TMEM175 deficiency provokes α-syn accumulation with defects in macroautophagy, lysosomal degradation, and mitochondrial respiration processes [117].

Another lysosomal protein with significant links to PD is the transcription factor EB (TFEB), which coordinates expression of lysosomal hydrolases, membrane proteins, and genes involved in autophagy. This master regulator of lysosome biogenesis is regulated by mammalian target of rapamycin (MTOR)C1 through phosphorylation of specific serine residues [180]. A study based on a rodent model of α-syn-induced toxicity confirmed that the autophagy-lysosomal pathway is impaired, with TFEB retained in the cytosol. Therefore, increasing autophagy-mediated degradation of SNCA via TFEB regulation could be a promising strategy for PD prevention and treatment [180,181,182,183]. Several direct and indirect TFEB agonists have been described as potent regulator in pre-clinical and clinical trials [183]. 

Taken together, these observations lead us to conclude that any drugs that increase the functional properties of lysosomes should have a potent effect, halting the progression of PD.

### 3.5. Is the Autophagy Machinery a Potential Target for Selective Intervention in PD?

**Figure 4:**
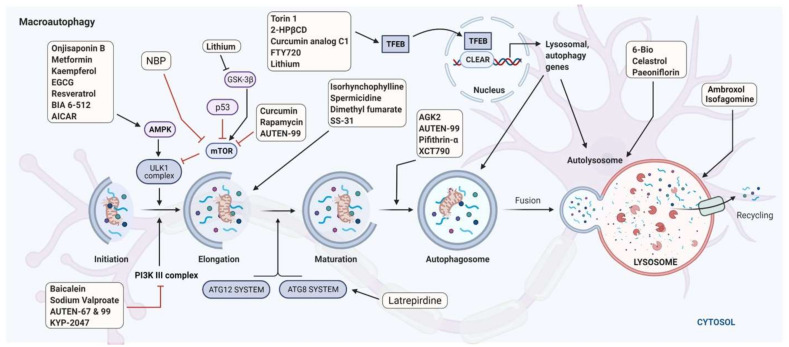
The macroautophagy pathway and potential drug targets for PD treatment. The diagram includes current small therapeutics and drug candidates (small molecules and peptides) that act at different stages of the macroautophagy pathways and might be beneficial in PD.

The link between PD and autophagy dysfunction still remains largely unknown. Thus, there is insufficient information to answer the question of whether autophagy alteration occur in all patients with PD, and is not associated only to PD risk genes. In this context, it would be of great interest to examine the autophagic activity at the cellular level in patients with and without mutations in autophagy-linked genes. Despite the complexity of the autophagy defects in PD, this vital cellular system could represent a target of considerable interest for the treatment of the disease. Indeed, to date, although our understanding of the molecular bases of PD and its diagnosis [184,185] are improving, therapeutic aspects of PD remain below expectations, with a limited arsenal of efficient and specific drugs. Thus, current therapies are essentially symptomatic, aiming to partially maintain dopamine levels by limiting its degradation using monoamine oxidase B inhibitors, supplying dopamine precursors using levodopa [186] or dopamine agonists such as ropinirole, pramipexole, or rotigotine [187] (Figure 4). Today, numerous strategies, including regenerative medicine-based solutions—such as cell-based implants, fetal grafts, patient-specific tissue-engineered constructs—and some cutting-edge technological approaches involving the delivery of near-infrared light directly into the SN in the brain of PD patients, have been used or are being explored [188,189]. Biologics, such as antibodies to α-syn (prasinezumab, developed by Roche and Prothena) have been evaluated but unfortunately demonstrated limited success in a first phase II clinical trial. Other companies are also exploring the same line of possible intervention with monoclonal antibodies to α-syn [190]. However, this line of treatment remains uncertain; in February 2021, the failure of Biogen’s cinpanemab was announced—this monoclonal antibody has a mode of action similar to Roche’s prasinezumab. In parallel, novel pharmacological interventions are being evaluated in clinical trials. Some of these also target α-syn [191] whereas others target TNF, transcription factors, nuclear factor erythroid 2–related factor 2 (NRF2), and PPARγ, G protein-coupled receptors, glucocorticoid receptors, glucagon-like peptide 1 (GLP1), and inflammasome/NLR family pyrin domain-containing 3 (NLRP3) (see [187,192,193,194]). Microglial NLRP3 is a source of sustained neuroinflammation that can contribute to progressive DA neuron loss [195]. Molecules and biologics targeting B and T lymphocytes are also being investigated. In this context, compounds targeting autophagy remains an unexplored route for the development of innovative treatments for PD.

A number of proof-of-concept studies have been performed with compounds targeting autophagy in vitro in cells and in vivo, generally in genetically-deficient animal models. The molecules tested were designed to target mitophagy, macroautophagy, CMA, or lysosomes (Table 3 [196]). In the context of mitophagy, since oxidative stress is one of the principal causes of mitochondrial dysfunction, the agents assessed mainly protect against the production of, or neutralize, free radicals. In addition, agents targeting mitochondrial biogenesis, such as nuclear respiratory factor 1 and 2 (NRF1, NRF2), TFAM, and PGC1-α (described above) have been investigated (Table 3). This process could also be controlled by tuning a few of its crucial interactions. For example, the tumor suppressor protein p53 is a relevant target as it interacts with PRKN, inhibiting its translocation to the cytosol. Compared to healthy controls, significantly higher levels of p53 protein have been measured in the caudate nucleus in PD patients [197]. Experiments in PD models effectively showed that the inhibition of this interaction activates PRKN-dependent mitophagy and reduces the symptoms of PD [197]. Several other targets linked to the mitophagy pathway have been, or are currently being, explored in relation to PD [197,198,199]. Emerging promising molecules include selective inhibitor of the mitochondrial deubiquitinase, USP30 that negatively regulates PRKN-mediated mitophagy [132,200]. In addition, agents that target mitochondrial dysfunction rather than mitophagy per se (e.g., nimodipine or tetrahydroisoquinoline; Table 3) were found to have a protective effect against PD [201,202].

Independent studies on animal models have also demonstrated that enhancing macroautophagy by acting on TFEB or BECN1 can protect neurons against α-syn-induced toxicity [180,275]. Nitolinib, anbroxol, curcumin, spermidine, Torin1, 2-hydroxypropyl-β-cyclodextrin (2-HPβCD), or the well-known excipient trehalose are all representative of this pharmacological class (Table 3 and Table 4) [196,276]. An important molecule to mention here is the Tat-Beclin-1 peptide, a cell-permeable peptide consisting of BECN1 (residues 267–284) conjugated to the HIV-1 Tat protein. This peptide construct enhances autophagy initiation by interacting with the autophagy inhibitor Golgi-associated plant pathogenesis-related protein 1 (GAPR-1/GLIPR2). This interaction leads to BECN1 distribution throughout the cytosol while also increasing autophagosome formation in neurons. Analogues of this peptide are currently being explored in clinical trials.

Molecules that target CMA are also highly relevant. The decisive role of LAMP2A in α-syn degradation has been clearly demonstrated (see above). Cells and *Drosophila* overexpressing LAMP2A display a capacity to resist α-syn-induced neurotoxicity or neuronal degeneration and PD-related features [293,294]. These data along with the data presented above, undoubtedly indicate that CMA regulators LAMP2A and HSPA8 represent targets of choice for PD treatments [192,295]. Geranylgeranylacetone, a nontoxic acyclic isoprenoid compound that has been clinically applied as an antiulcer drug in Asian countries, and is a known inducer of HSPs acting via the activation of heat shock transcription factor-1, phorbol 12-myristate 13-acetate and others, could represent exploratory molecules to treat PD via modulation of the CMA pathway [296].

As indicated above, numerous molecules that target mitophagy or lysosomal autophagy pathways are under investigation in preclinical or clinical studies (Table 3 and Table 4). To the best of our knowledge, however, none of them have yet been approved and/or are already applied in the treatment of PD.

## 4. Awaiting Satisfactory Answers—Future Research

A significant number of new research lines suggest novel avenues of investigation centered on autophagy. In the specific context of PD, we reviewed above some of the key results indicating that targeting the mitophagy and CMA pathways could be a means to protect against α-syn-related toxicity (Appendix B provides a general significance statement). However, some limitations remain, related both to the development of efficient pharmacological molecules, to their administration, and to some theoretical considerations.

In fact, very few molecules are selective to one type of autophagy, or even autophagy as distinct from other cellular pathways, such as apoptosis [48,271,297,298,299,300,301] (Figure 4). Consequently, most molecules may display unwanted side-effects, especially when they are administered daily in the medium- or long-term to PD patients. Stability of a compound can also represent a limitation for its use. In general, the half-life in the body is around 10–25 min, especially in the liver. It should be mentioned, however, that in most organs and tissues, induction of autophagosome formation is very rapid. Therefore, activation of autophagy remains a rational target strategy when aggregated proteins accumulate. In addition, autophagosomes generally recycle quickly. This represents an advantage with regard to possible toxic events linked to the consequences of induced/activated autophagy. However, it can also be a limitation, in the sense that it will be necessary to extend the treatment periods. In fact, in the particular case of neurons, autophagosome biogenesis is extremely complex, and some heterogeneity has been described depending on the neuronal compartment considered. This aspect remains a matter of debate. Further in vivo information is also required regarding autophagosome formation and dynamics in developing versus mature systems [302].

Autophagy is a very dynamic compartmentalized process; it can be increased in certain organs and tissues but decreased in other organs in the same subject. This differential activation has been described in several models of chronic inflammation and autoimmune diseases, for example in murine models of Sjögren’s syndrome [303], chronic inflammatory demyelinating polyneuropathy [304] and chronic house dust mite-induced airway inflammation [305]. With regard to neurons, as indicated above, autophagy is even more complex, with specific stages of the pathway occurring in distinct subcellular compartments. As a consequence, treating an individual with a compound to induce or inhibit autophagy can have a range of individual effects. Due to this variety of effects, treating a subject with an inhibitor can restore abnormally weak levels of autophagic activity in another tissue, as illustrated by the effect of the CMA-modulator peptide P140 [303,304,305]. P140 that targets CMA and probably indirectly macroautophagy, has shown correcting effect on altered CMA activity, but display no effect on the basal, well balanced and vital autophagy process. This therapeutic peptide is currently evaluated in phase III clinical trials for lupus. 

Another critical question that arises is the timing of treatment with regard to the course of the disease. How early should we intervene to see efficacy? This aspect has not really been solved and raises the general question of the benefit-risk of such treatments. The functionality of autophagy that declines with age also represents an aspect that must be taken into consideration in any autophagy-based treatment of PD.

## 5. General Conclusions

Although the considerations described in this review highlight some gaps in our understanding and appreciation of the potential of autophagy modulators to treat PD, several molecules hold promise for future specific treatment. An important aspect to underline here is that these molecules act on a cellular mechanism and not on the final damage induced. They could therefore be included in early treatment, or even as part of preventive strategies, to avoid or halt disease development.

Interestingly, in addition to the molecules described above in the context of PD, others that target autophagy have shown beneficial effects against neurodegenerative diseases [306,307,308,309], and could possibly be considered for review of their indications. These include, for example, neferine, Lu AE58054/idalopirdine, SB-742457, latrepirdine, MCI-186/Edaravone, SAGE217, GSK621, AICAR, Propofol, A769662, RSVA314, RSVA405, AUTEN-67, cystatin C, MSL, Digoxin, FTY720, carbamazepine, rilmenidine, clonidine, verapamil, SMER28, BRD5631, and AUTEN-67, among others. However, their selectivity, efficacy, and safety must be demonstrated in the context of PD. Extensive work is being undertaken to discover potent new chemical compounds for PD treatment [308,310]. Novel targets that are closely linked to autophagy pathways could also prove to be relevant in PD, for example, protein-O-linked N-acetyl-β-D-glucosaminidase (O-GlcNAcase) [311,312].

From a technical point of view, it is worth recalling here that it is highly recommended to study the efficacy of these novel strategies in several independent models, both in vitro and in vivo if we hope to achieve reproducible results and, in the context of autophagy, to monitor several relevant biomarkers [313,314,315], as well as measuring the autophagic flux [79,80].

PD affects 1–2 individuals per 1000 in the general population at any time. Its prevalence increases with age. In industrialized countries, it is estimated that the disease affects 0.6%–0.8% of 65–69-year-old individuals and 2.6%–3.5% of 85–89-year-olds. Currently, no specific test exists to diagnose PD, and it cannot be cured. Medications (dopaminergic drugs) as well as surgical treatment only act on symptoms. The ultimate goal of ongoing investigations is to develop, ideally non-invasive, therapies that could retune the cellular degradation pathways responsible for clearing abnormally folded or aggregated proteins that are toxic for neurons. Targeting autophagy without altering other vital cellular pathways is a challenge that may be achievable in PD and other neurodegenerative diseases if safe and selective molecules can be appropriately applied and delivered.

## Figures and Tables

**Table 1:** Types of selective autophagy.

Processes	Organelles and Substrates	Selective Markers	Functions
Aggrephagy	Protein aggregates	MAP1LC3, Alfy	In addition to the proteasome and CMA—which degrade misfolded and unwanted soluble proteins—this process degrades protein aggregates [37].
Crinophagy	Secretory vesicles or granules	HOPS	Degrades secretory vesicles and granules, which evade the innate process of exocytosis [38,39]. Unnecessary or obsolete secretory granules directly fuse with late endosomes/lysosomes allowing rapid elimination of unused secretory material from the cytoplasm. Discovered in 1966. Remains little known.
CMA	Proteins that contain KFERQ-motif	LAMP2A, HSPA8	Unlike macroautophagy and microautophagy, CMA sequesters soluble proteins with KFERQ-motif in a HSPA8- and LAMP2A-dependent manner [40].
DNautophagy	DNA	LAMP2C	Degrades DNA via LAMP2C in an ATP-dependent manner [41].
ER-phagy (also referred to as reticulophagy)	Endoplasmic reticulum	Syntaxin 17, DFCP1	Unlike UPS, which is dedicated to the degradation of ER proteins, this process clears unwanted membrane lipids and protein aggregates [42]. Is involved in the maintenance of protein folding, processing, and transport, lipid and steroid synthesis, calcium storage, and detoxification. Half-life: 3–5 days.
Ferritinophagy	Ferritin	NCOA4	Involved in the degradation of ferritin (iron protein complex), when cellular iron levels are low [43].
Glycophagy	Glycogen	STBD1/Genethonin-1	Process in which cellular glycogen is delivered to the vacuole and degraded in response to changing cellular conditions. Plays a crucial role in maintaining glucose homeostasis in many tissues, including heart, liver and skeletal muscles [44]. Is essential for lipid droplet biogenesis.
LAP	Bacteria, living and dead cells	Lipidated LC3	A recently discovered form of non-canonical autophagy, in which receptor engagement during phagocytosis triggers the recruitment of certain (not all) members of the autophagy machinery to the single-membrane cargo-containing phagosome, or LAPosome. Degrades pathogens, dying cells, or antibody-coated particles recognized thanks to TLR, phosphatidylserine, and Fc receptor, respectively. The engulfment of the Fc receptor triggers recruitment of the RUBICON-containing Class III PI3K complex to the cargo-containing phagosome. First described in 2007 [45].
Lipophagy	Lipid droplets	LIPA	Degrades lipid droplets, a unique structure surrounded by a phospholipid monolayer that separates neutral lipids from the cytoplasmic environment. Very short half-life [46].
Lysophagy	Lysosomes	LAMP2A	Degrades highly permeabilized or damaged lysosomes [47]. Lysosomes are double membrane-bound acidic organelles, which degrade unwanted materials received via endocytic, phagocytic, and autophagic pathways. They play significant roles in nutrient-sensing and cholesterol homeostasis [48].
Mitophagy	Mitochondria	PINK1, RKN	Degrades mitochondria [49]. Half-life: 14 days (heart), 2 days (liver).
Nucleophagy	Nucleus	ATG39 *	Degrades nucleus-derived material [50,51]. In mammalian cells, nucleophagic activity is linked to oncogenic and genotoxic stress.
Pexophagy	Peroxisomes	ATG36	Degrades peroxisomes—membrane-bound organelles playing an essential role in a variety of metabolic reactions (e.g., purine catabolism, fatty acid beta-oxidation, bile acid synthesis, ether phospholipid synthesis) [52]. Half-life: 5 days.
Proteaphagy	Proteasomes	Proteasome (depends on the size of the proteasome)	Degrades proteasomes and is therefore involved in the regulation of proteasome turnover [53,54]. Discovered in 1995 when proteasomes were observed within autophagic vesicles and lysosomes in rat liver cells under starvation conditions [53,54], confirmed and termed “proteaphagy” in 2015 [55]. Half-life: 12–15 days.
Ribophagy	Ribosomes	Ubp3, Bre5, Rsp5	Degrades ribosomes containing RNA and ribosomal proteins, which decode the genome and are involved in the formation of peptide bonds [56]. The composition of the ribosome pool is heterogenous; numerous inherent ribosome properties can promote preferential translation of distinct cellular mRNAs. Half-life: 5–10 days.
RNautophagy	RNA	LAMP2C	Degrades RNA via LAMP2C in an ATP-dependent manner [57].
Synucleinphagy	α-syn	SQSTM1	Degrades α-syn. Microglia ingest and degrade α-syn released by neurons via SQSTM1-mediated selective autophagy. Shown both in vivo and in vitro. The process requires the presence of TLR4, which interacts with α-syn to induce the transcriptional upregulation of Sqstm1 through the NFKB pathway [58].
Xenophagy	Invading microbes	CALCOCO2/NDP52	Degrades intracellular pathogens (bacteria, protozoans, and viruses) that are either present in the cytosol or contained in pathogen-containing vacuoles.
Zymophagy	Pancreatic zymogen granules	VMP1	Degrades zymogen granules. VMP1, the ubiquitin-protease USP9x, and SQSTM1 mediate the zymophagy process. VMP1 is a multispanning transmembrane protein associated with the ER and Golgi complex that participates in autophagosome formation [59,60]. Although VMP1 is upregulated in acute pancreatitis, it is essential for autophagosome formation [60].

* Yeast-specific receptor; no specific receptor has been identified in mammals. Abbreviations not described in the text: Alfy, PI3P-binding Autophagy-linked FYVE domain protein; ATP, adenosine-triphosphate; Bre5, Ubp3-associated cofactor; CALCOCO2, calcium-binding and coiled-coil domain 2; DFCP1, double FYVE domain-containing protein; HOPS, homotypic fusion and vacuole protein sorting; LAMP2C, lysosomal-associated membrane protein 2C; LIPA, lysosomal acid lipase A; NCOA4, nuclear receptor coactivator 4; Rsp5, E3 ubiquitin ligase; STBD1, Starch-binding domain-containing protein 1; Ubp3, ubiquitin-specific protease 3; VMP1, vacuole membrane protein 1.

**Table 2:** Genetic mutations linking autophagy components to PD *.

Genes	Functions	Link to PD
*ATG5*	ATG5-dependent autophagy protects various cells, including neurons, from apoptosis [99].	Reduced expression of ATG5 and its variants is implicated in PD pathology, and increased expression reverses PD pathology [100].
*ATG7*	ATG7 is a vital part of the ATG8 and ATG12 conjugating systems of autophagy, and plays an essential role in neuronal development [101].	Four ATG7 gene promoter variants have been identified and implicated in the development of sporadic PD [102].
*ATG12*	In conjunction with other ATGs, it forms an ATG12 system, which is essential for autophagosome formation.	Three ATG12 gene promoter variants have been identified and implicated in the development of sporadic PD [103].
*ATP6AP2*	Vacuolar ATPase localized on the lysosomal membrane that regulates pH.	Exon skipping mutations in *ATP6AP2* strongly associated with PD. Mutation leads to impaired lysosomal acidification [104,105].
*ATP10B*	P4-type ATPase present on the late endo/lysosomal compartment. In addition to its lipid flippase function (transfer of lipids from the exoplasmic to the cytoplasmic membrane leaflet), it also transports cell cycle regulator proteins from the ER to endosomes and lysosomes [9,13].	Knockdown of *ATP10B* in cortical neurons elevated the lysosomal pH and lysosomal mass, thereby reducing lysosomal degradative function [13].
*ATP13A2/PARK9*	P5-type ATPase localized on vesicular structures, particularly lysosomes; functions as a cation transporter [10].	Closely linked to Kufor–Rakeb syndrome and YOPD [10].
*DJ-1/PARK7*	Functions as an antioxidant and chaperone. Through its diversified functions, it maintains mitochondrial homeostasis [106].	Strongly associated with YOPD. Lack of *DJ-1* leads to reduced mitochondrial membrane potential and increased autophagy markers [106,107,108].
*DNAJC13/PARK21*	Retromer-mediated endosomal protein sorting [106].	Closely linked with late-onset of PD [106].
*FBOX7/PARK15*	In association with PINK1 and PRKN, regulates mitophagy [109].	Closely linked to impaired mitophagy. Mutation leads to autosomal recessive YOPD [109].
*GBA1*	GBA is a lysosomal enzyme, which degrades cell membrane glycolipids, i.e., glycosylceramide [48].	Heterozygous defects linked to PD. Mutation impairs autophagic-lysosomal function [110,111]. Lack of GBA activity causes Gaucher’s disease [48].
*HSPA8*	Acts as a substrate carrier protein in CMA.	In PBMCs, decreased transcript levels observed in PD patients [88].
*LRRK2*	LRRK2 is known for sorting various vesicles, based on its interactions with Rab5b, Rab7, Rab7L1, Hsc70, and others [10].	Strongly associated with late-onset PD. Like α-syn, variants of the *LRRK2* locus have been observed [10]. Increased kinase activity of LRRK2 and reduced autophagic flux were observed [112,113].
*MAP1LC3*	MAP1LC3B and its subfamily members are crucial to autophagosome formation, elongation and maturation [88].	Although mutations are not reported, significant increases in expression of ATG8 subfamily genes are observed [88].
*PRKN/PARK2*	Gene that encodes PRKN (E3 ligase), which plays an essential role in mitophagy in parallel with PINK1.	Mutations in this gene impair mitophagy, lead to autosomal recessive and sporadic early-onset PD [114,115]. Responsible for dominant forms of PD.
*PINK1/PARK6*	Member of the serine-threonine kinases class, plays a crucial role in mitochondria quality control by inducing mitophagy.	Closely linked to mitophagy impairment Mutation leads to autosomal recessive and sporadic YOPD [116].
*TMEM175*	Gene that encodes endo/lysosomal K^+^ channel [117].	Regulates K^+^ conductance on endo/lysosomal membranes to maintain membrane potential, pH stability, and regulate organelle fusion [117].
*TMEM230*	Gene that encodes transmembrane protein involved in retromer and secretory vesicle trafficking functions.	Mutations alter trafficking function [118].
*VPS13C/PARK23*	Mitophagy development.	Closely linked to YOPD [4].
*VPS35/PARK17*	As a part of the retromer complex, it performs protein trafficking functions [106].	Linked to late-onset PD [106].

* More information about the genetics of PD can be found in the following references [4,7,8,9,10,11,63,106]. Abbreviations not used in the text: Fbxo7, F-box only protein 7; Rab, Ras analogue in the brain.

**Table 3:** Therapeutic autophagy-targeting molecules with potential in PD *.

Candidate	Mechanism of Action	Comments
AGK2	Enhances autolysosome formation	In Vitro studies on yeast (Saccharomyces cerevisiae) and mammalian cells (HeLa) confirmed its effect on clearance of α-syn aggregates [203].
AUTEN-99 (autophagy enhancer-99)	Inhibits the phosphatase activity of MTMR14 (Jumpy), a negative regulator of autophagy	Unlike other major autophagy enhancers, it actively crosses the blood-brain barrier [204]. Preliminary results on Drosophila model confirmed efficacy against neurodegenerative disease, including PD [204].
Baicalein	Induces PI3K-mediated autophagy	Classical flavonoid from Scutellaria baicalensis (Lamiaceae) with wide pharmacological actions. Its autophagy-inducing effect has been widely explored in cancer and neurodegenerative diseases [205,206]. It covalently binds to α-syn and prevents its transmission via complex formation and autophagy activation [207]. Though conflicting results of Baicalein on autophagy exist, it is reported to be effective against PD [208,209,210].
6-Bio	Enhances autolysosome formation in DA neurons	Preclinical studies in mouse models showed effects on clearance of α-syn aggregates [211].
Celastrol	Enhances autolysosome formation	Sourced from *Tripterygium wilfordii* (Celastraceae). In vitro, data on neuroblastoma cells confirmed enhancement of autophagy and clearance of α-syn aggregates [212].
Curcumin	Inhibits MTOR/p70S6K	Extracted from turmeric, a natural spice that gives curry its yellow color. Chronic dietary supplementation with curcumin in α-syn transgenic mice resulted in reduced motor defects. It also increased α-syn phosphorylation, which increases α-syn degradation [213]. In Vitro studies on SH-SY5Y cells (expressing A53T α-syn) confirmed that α-syn accumulation was reduced and autophagy was induced [214].
Curcumin analogue C1	MTOR-independent TFEB enhancer. Selectively binds to the N-terminus of TFEB and promotes its nuclear translocation	Protects neuronal cells from 6-hydroxydopamine/ascorbic acid-induced toxicity; alleviates symptoms in PD mice [182,215].
Dimethyl fumarate	Modulator of SQSTM1-dependent autophagy	Activates the master regulator functions of NRF2, an antioxidant transcription factor, which rescues α-syn aggregate-induced neuronal death via SQSTM1-dependent autophagy activation [216]. Requires confirmation.
Epigallocatechin gallate (EGCG)	Activates AMPK	Induces autophagy in neuronal cells [217]. Protects DA neurons against death by inhibiting mitochondrial dysfunction in both mutant LRRK2 and PRKN-null Drosophila models [218].
2-HPβCD	Activates TFEB	Significantly enhances degradation of TFEB-mediated α-syn aggregates in human neuroglioma cells [219].
Isofagomine	GCase	Like Ambroxol, it is also a molecular chaperone for GCase [220]. Studies on human fibroblasts from PD patients with *GBA* mutations and *Drosophila* expressing mutant forms of *GBA* showed enhanced GCase activity [221]. Reduces ER stress and improves motor function [58].
(Iso)rhynchophylline	Activation of BECN-mediated autophagy	A tetracyclic oxindole alkaloid extracted from the Chinese herbal medicine Uncaria rhynchophylla (Miq.) Jacks. Therapeutic potency promoting α-syn aggregate clearance has been documented both in in vitro (N2a, SH-SY5Y and PC12 cells, and primary cortical neurons) and in vivo (Drosophila) models [103,222,223].
Kaempferol	AMPK/MTOR-mediated pathway	A natural dietary flavonoid with potent chemopreventive effect [224]. Studies performed on SH-SY5Y cells, primary neurons, and striatal cells showed a protective effect against rotenone-induced toxicity. This effect was mediated through the activation of mitophagy [225].
KYP-2047	Prolyl endopeptidase inhibitor III	Inhibits the formation of α-syn aggregates both in mutant cells (A30P/A53T mutant human α-syn) and in the brain of transgenic mice (A30P α-syn strains) [226]. This effect was mediated though the induction of BECN1-mediated autophagy [227,228].
Latrepirdine (Dimebon; dimebolin)	Enhances *ATG8*-dependent autophagy	Currently evaluated in clinical trials against AD and HD [229]. Chronic treatment abrogated α-syn accumulation in mouse brain [229].
Lithium	Reduces IP_3_ production by inhibiting inositol monophosphatase	As a mood stabilizer, lithium has shown a neuroprotective effect in various neurodegenerative models, including PD [230,231,232,233]. In addition to effects on autophagy, it also inhibits GSK-3β, either by interfering with catalytic Mg2+ or by inducing its phosphorylation at serine 9. Upregulation of GSK-3β has been implicated in AD [234]. Both effects are dose-dependent; lower doses activate autophagy, whereas higher doses inhibit autophagy. Higher doses inhibit GSK-3β better than lower doses [231].
MCC950	Potent, selective inhibitor of NLRP3 with IC50 of 7.5 nM in BMDMs	Although not well explored in neurons, an enhanced effect of MCC950 on autophagy has been documented [235,236,237]. Exhibits anti-inflammatory activity on microglial neuroinflammation, mediated by fibrillar α-syn-induced NLRP3 activation [238]. Beneficial effect confirmed in mouse models of PD [238].
Metformin	Antihyperglycemic agent of the biguanide class. Acts via both AMPK-dependent and AMPK-independent mechanisms	Approved by the U.S. FDA as a prescription medication to treat diabetes. Studies performed in vitro (SH-SY5Y cells) [239,240,241] and in vivo, using drosophila [218] and mouse [240,242] models showed protective effects against PD.
Onjisaponin B	Triggers autophagy via the AMPK/MTOR-mediated signaling pathway	A triterpenoid saponin from *Radix Polygalae* (Polygalaceae). In Vitro studies have shown that it clears mutant α-syn in rat pheochromocytoma cells (PC-12 cells) [243].
Paeoniflorin	Enhances autolysosome formation	A monoterpene glucoside extracted from Paeoniae alba (Paeoniaceae) [244]. Contributes to the degradation of mutant α-syn in PC-12 cells by modulating acid-sensing ion channels and autophagic machinery [245].
Pifithrin (PFT)-α	Specific inhibitor of p53 transcription activity. Displays potent p53-independent activity in cells.	PFT-α and its analogue PFT-μ reduce mRNA levels of traumatic brain injury (TBI)-induced pro-inflammatory cytokines (IL-1β and IL-6), as well as modulating localization of TBI-induced autophagic markers (MAP1LC3 and SQSTM1) [246]. Treatment with PFT-μ mitigates TBI-induced reductions in mRNA levels of PINK-1 and SOD2. Treatment with PFT-α in the MPTP-induced PD mouse model rescues neuronal cell death [197,247]. PFT-μ also showed a beneficial effect in PD (requires confirmation) [248]. PFT-α-mediated mitophagy activation is also effective in type I and II diabetic mouse models [249].
Rapamycin and its derivatives (CCI-779, RAD001 and AP23573)	Inhibitors of MTOR	The autophagy enhancer rapamycin and its analogues have shown promising efficacy in various models of PD [250,251,252,253,254,255].
Sodium valproate	Synergistic to lithium-induced autophagy	Combination of valproate with lithium has shown therapeutic efficacy in the MPTP mouse model of PD, where it enhances autophagy [256]. Despite the broad use of valproate to treat several psychiatric and neurological disorders, this molecule has only a slight or no effect in PD [257,258]. However, it induces a reversible form of parkinsonism effects [259,260].
Spermidine	Autophagy inducer that maintains cellular and neuronal homeostasis. May achieve effects via BECN1 and TFEB-mediated pathways	A naturally occurring endogenous polyamine synthesized from diamine putrescine. Levels decline with age. Supplementation with spermidine has shown protective effects against motor dysfunction, neuronal loss in Drosophila melanogaster and Caenorhabditis elegans expressing human α-syn [261]. Spermine synthesis and its metabolite, N1,N8-diacetylspermidine, may be useful diagnostic and severity biomarkers for PD, respectively [262].
SS-31 (D-Arg-2′6′-dimethylTyr-Lys-Phe-NH2)	BECN1-mediated autophagy	A mitochondria-targeted tetrapeptide that belongs to the SS peptide family. Designed to localize to the mitochondria and inhibit free radicals. Showed protective effects on DA neurons in MPTP mouse model [263,264]. Corrects dysregulated autophagy in type 2-diabetes [265].
Torin1	MTOR-dependent TFEB enhancer	Protects neuronal cells from 6-hydroxydopamine/ascorbic acid-induced toxicity and alleviates symptoms in PD mice [182,215].
Trehalose	Induces autophagy via lysosome-mediated TFEB activation	Extensively used as an excipient [266]. Recent findings revealed that it activates autophagy [252,267]. Contributes to the clearance of α-syn aggregates [268].
MF-094	Selective USP30 inhibitor	Treatment with MF-094 has shown potent protein ubiquitination and accelerated mitophagy in vitro [132,200,269].
XCT790	Modulates autophagosome formation by estrogen-related receptor α-dependent manner	Contributes to the clearance of α-syn aggregates and corrects some motor coordination defects [270].

* Further information about drug candidates or drug-like molecules that have potential in PD via their effects on autophagy processes can be found elsewhere [103,271,272,273,274]. Abbreviations not used in the text: BMDMs, bone-marrow-derived macrophages; GCase, β-glucocerebrosidase; GSK-3β, glycogen synthase kinase-3 beta; IP3, inositol-1,4,5-trisphosphate; MPTP, 1-methyl-4-phenyl-1,2,3,6-tetrahydropyridine; MTMRs, myotubularin-related phosphatases; SS, Szeto–Schiller, USP, ubiquitin specific peptidase.

**Table 4:** Therapeutic autophagy-targeting molecules under clinical evaluation for use in PD *.

Drugs	Characteristics and Mechanism of Action	Stage of Development/Sponsors	Identifiers
Ambroxol	Well-known expectorant and mucolytic agent. In addition to its diverse functions, also acts as a potent chaperone for GCase [277]. In Vitro and in vivo studies in wild-type and transgenic models indicate that its therapeutic effects in the context of PD involve modulating the autophagy process [221,278,279]. These effects are mediated by the TFEB pathway and through increased exocytosis [280]. A recent clinical study in PD patients with and without *GBA1* mutations provided positive preliminary data for large cohort studies [281].	Phase II/University College, London, UK (NCT02941822), Lawson Health Research Institute (NCT02914366)	NCT02941822; NCT02914366
BIA 6-512 (trans-resveratrol)	An analogue of a natural compound, resveratrol, which has a wide pharmacological action, especially in PD [282,283]. Potential antioxidant; it also initiates Sirtuin 1 (SIRT1)-dependent AMPK activation [103,283].	Phase I/Bial—Portela C S.A.	NCT03095092
Coenzyme Q10 (CoQ)	Free radical scavenger used as a supplement in PD. Reduced CoQ in cells triggers mitophagy [284].	Phase III/Weill Medical College of Cornell University (NCT00740714), Technische Universität Dresden, Germany (NCT00180037)	NCT00740714; NCT00180037
DL-3-n-butylphthalide (NBP)	An active component isolated from *Apium graveolens* (Apiaceae) [285]. Its antioxidant function, and capacity to correct mitochondrial dysfunction is widely documented [286]. Approved by the Chinese FDA for the treatment of ischemic stroke [287]. Various preclinical models of PD have confirmed its efficacy in alleviating neuronal toxicity via autophagic mechanisms; indirectly inhibits MTOR-mediated autophagy [286,288,289].	Phase II/First Affiliated Hospital of Bengbu Medical College (Bengbu, China)	ChiCTR1800018892
MitoQ (mitoquinone)	A conjugate of lipophilic triphenylphosphonium cation and coenzyme Q10. Powerful antioxidant. Studies with MitoQ on leukemia cells (HL-60) and hepatocellular carcinoma cells (HepG2) confirmed the induction of autophagy following activation of AMPK and inhibition of MTOR [207].	Phase II/Antipodean Pharmaceuticals, Inc.	NCT00329056
Nicotinamide riboside	Used as a supplement. Nicotinamide (in various forms) induces autophagy by acting on SIRT1-dependent and MTOR-mediated mechanisms [290,291].	ND/Haukeland University Hospital, Norway	NCT03816020
Nilotinib	An Abelson kinase/tyrosine kinase inhibitor. Approved by the US FDA (2007) for the treatment of chronic myelogenous leukemia. Preclinical studies on transgenic mice models (A53T mutation of human α-syn) have shown that it increases α-syn clearance via BECN1-mediated autophagy [292]. Reduces cell death and improves motor function. Under evaluation for treatment of other neurodegenerative diseases (AD, NCT02947893; HD, NCT03764215).	Phase II/Georgetown University, USA	NCT02954978

* Updated: June 2021. Abbreviations not used in the text: FDA, Food and Drug Administration; GCase, β-glucocerebrosidase; ND, not disclosed.

## Data Availability

Not applicable.

## Abbreviations

α-syn, α-synuclein; Aβ, amyloid beta; AD, Alzheimer’s disease; ALS, amyotrophic lateral sclerosis; AMPK, AMP-activated protein kinase; APP, amyloid precursor protein; ATG, autophagy-related; AV, autophagic vacuole; BECLIN1/BECN1, B-cell lymphoma 2 interacting myosin/moesin-like coiled-coil protein 1; CMA, chaperone-mediated autophagy; CNS, central nervous system; DA, dopaminergic; eMI, endosomal microautophagy; ER, endoplasmic reticulum; HD, Huntington’s disease; LAMP2A, lysosomal-associated membrane protein 2A; LAP, LC3-associated phagocytosis; LB, Lewy body; LN, Lewy neurite; LRRK2/dardarin, leucine-rich repeat kinase; MAP1LC3, microtubule-associated protein 1A/1B-light chain 3; MTOR, mammalian target of rapamycin; PD, Parkinson’s disease; PE, phosphatidylethanolamine; PI3K, phosphatidyl inositol 3-kinase; PINK1/PARK6, PTEN-induced putative kinase 1; PRKN, PARKIN; SMAC, single membrane ATG8 conjugation; SN, *substantia nigra*; SNpc, SN *pars compacta*; SQSTM1/p62, sequestosome 1; TLR, Toll-like receptor; ULK1, Unc-51-like kinase 1; UPS, ubiquitin-proteasome system; YOPD, young-onset PD.

## Appendix A

**Alzheimer’s disease (AD)**

The most common cause of dementia, a general term for loss of memory and other cognitive abilities serious enough to interfere with daily life. AD accounts for 60–80% of dementia cases.

**Amyotrophic lateral sclerosis (ALS)**

ALS (also known as motor neuron disease or Lou Gehrig’s disease) generally starts with muscle twitching and weakness in one limb, or slurred speech. It can affect control of the muscles needed to move, speak, eat, and breathe, and can be fatal.

**Autophagosomes**

A double membrane-bound vesicle, which encloses cellular constituents and fuses with lysosomes to form phagolysosomes where the engulfed material is digested or degraded and either released into the extracellular matrix via exocytosis, or released intracellularly to undergo further processing.

**Autophagy**

A vital, finely-regulated and evolutionarily-conserved intracellular pathway that continuously degrades, recycles, and clears unnecessary or dysfunctional cellular components. Autophagy is crucial for cellular adaptation to the environment and to maintain cell homeostasis, especially under stress conditions.

**Chaperone-mediated autophagy (CMA)**

Selective autophagy pathway in which proteins containing a KFERQ-like sequence are captured by the HSAP8 chaperone and translocated into lysosomes following interaction with LAMP2A, which acts as a rate-limiting factor in this process.

**Endocytosis**

A vesicle-mediated process by which cells engulf membrane and extracellular materials. Several endocytic pathways—phagocytosis, pinocytosis, and receptor-mediated endocytosis—use distinct mechanisms to internalize material. Clathrin-mediated endocytosis (CME) is the major endocytic pathway in mammalian cells.

**Endosomal-lysosomal system**

A set of intracellular membrane-delimited compartments that dynamically interconvert from early endosomes, recycling endosomes, late endosomes, and the lysosome.

**GABARAP**

A member of the ATG8 family that also includes MAP1LC3. It interacts with the GABA_A_ receptor and tubulin, and promotes tubulin polymerization.

**Huntington’s disease (HD)**

A progressive brain disorder that causes uncontrolled movements, emotional problems, and loss of thinking ability (cognition).

**Isolation membrane/phagophore**

Small vesicular sac derived from the ER, mitochondria, vacuoles, and other unidentified sources, which sequesters and folds away a portion of the cytoplasm at the very first step of macroautophagy.

**LC3-associated phagocytosis (LAP)**

LAP combines the molecular machineries of phagocytosis and autophagy, resulting in conjugation of the autophagic marker MAP1LC3 to phosphatidylethanolamine (PE) on the phagosomal membrane, generating “LAPosomes” that undergo facilitated fusion with lysosomes.

**Lewy bodies (LB)**

Abnormal aggregates of α-syn and ubiquitin found in the cytoplasm of neurons from patients with PD. LB are initially present in dopaminergic neurons (*substantia nigra*), but as PD progresses they may also be found in cortical neurons. It is not fully understood how LBs contribute to neuronal cell death.

**Lewy neurites (LN)**

Aggregates of abnormal α-syn localized in axons. Like LBs, LNs may be found initially in the bulb, pons, and mesencephalon, but may extend later to the cortex.

**Lysosome**

An organelle located in the cytoplasm of eukaryotic cells, containing acid hydrolases, which can break down virtually any kind of biomolecule, including proteins, nucleic acids, carbohydrates, lipids, and cellular debris.

**Macroautophagy**

Finely-regulated process during which the cell forms a phagophore, a double membrane compartment sequestering its contents, which matures into an autophagosome.

**Microglia**

A type of small macrophage-like glial cell in the CNS

**Mitophagy**

A key process that selectively disrupts damaged mitochondria by autolysosomal degradation, preventing excessive production of reactive oxygen species, and activation of cell death.

**PARKIN (PRKN)**

A protein encoded by the human *PARK2* gene, the precise functions of which are unknown. PARKIN is a component of a multiprotein E3 ubiquitin ligase complex, present in the ubiquitin-proteasome system (UPS) that targets proteins for degradation.

**Phagocytosis**

An endocytic process by which certain cells known as phagocytes (e.g., macrophages) internalize large particles (>0.5 µm) including bacteria, other microorganisms, foreign particles, and aged red blood cells to form a phagosome.

**Proteasome**

Protein complexes containing proteases, which degrade unneeded or damaged proteins. They are found in cells from all eukaryotes and archaea, and in some bacteria. Each human cell contains about 30,000 proteasomes.

**Rab proteins**

During the autophagy process, several vesicular structures are formed, transported and degraded by the fusion with lysosomes. The proteins that helps in the vesicular trafficking are called Rab protiens. Rab proteins are small GTPases, which belongs to the Ras-like GTPase, play a critical role in regulating autophagy.

**SEQUESTOSOME 1(SQSTM1)**

SQSTM1, also known as the ubiquitin-binding protein p62, acts as a major cargo receptor during the sequestration process. It acts both in a MAP1LC3-dependent and -independent manner.

**α-SYNUCLEIN (α-syn)**

A 140-amino acid residue soluble protein mostly found in the presynaptic neurons. The gene *SNCA* encodes α-synuclein, and mutations of this gene cause familial PD.

**Ubiquitin-proteasome system (UPS)**

A major proteolytic system that controls protein degradation and regulates many cellular processes in eukaryotic cells, such as DNA repair, stress responses, and cell proliferation. The UPS consists of specific enzymes that modify protein substrates using ubiquitin, and 26S proteasomes responsible for proteolysis of ubiquitin-tagged substrates.

## Appendix B

**Significance Statement**

Autophagy plays an important role in the onset and development of neurodegeneration linked to the accumulation of misfolded proteins or dysfunctional mitochondria. Dysfunction of canonical and non-canonical autophagy processes are involved in neurodegenerative diseases, especially Parkinson’s disease (PD). Both genetic (ATG genes) and cellular autophagy defects have been described in young-onset and classical PD. Defects in the autophagy-lysosomal pathway in dopaminergic neurons may result in the excessive accumulation of damaged proteins in the cytoplasm, ultimately resulting in cell loss and the neuropsychiatric manifestations characteristic of PD. The rational introduction of specific agents to modulate mitophagy and other forms of lysosome-dependent autophagy processes, such as chaperone-mediated autophagy, could represent promising avenues for the treatment of PD.
